# Good places for ageing in place: development of objective built environment measures for investigating links with older people's wellbeing

**DOI:** 10.1186/1471-2458-11-839

**Published:** 2011-11-01

**Authors:** Elizabeth J Burton, Lynne Mitchell, Chris B Stride

**Affiliations:** 1 School of Engineering and School of Health and Social Studies (joint appointment), University of Warwick, Coventry, CV4 7AL, UK; 2 School of Health and Social Studies, University of Warwick, Coventry, CV4 7AL, UK; 3 Institute of Work Psychology, University of Sheffield, Mushroom Lane, Sheffield, S10 2TN, UK

## Abstract

**Background:**

There is renewed interest in the role of the built environment in public health. Relatively little research to date investigates its impact on healthy ageing. Ageing in place has been adopted as a key strategy for coping with the challenges of longevity. What is needed is a better understanding of how individual characteristics of older people's residential environments (from front door to wider neighbourhood) contribute to their wellbeing, in order to provide the basis for evidence-based housing/urban design and development of interventions. This research aimed to develop a tool to objectively measure a large range of built environment characteristics, as the basis for a preliminary study of potential relationships with a number of 'place-related' functional, emotional and social wellbeing constructs.

**Methods:**

Through a review of urban design literature, design documents, and existing measures, a new tool, the NeDeCC (Neighbourhood Design Characteristics Checklist) was developed. It was piloted, refined, and its reliability validated through inter-rater tests. A range of place-related wellbeing constructs were identified and measured through interviews with 200 older people living in a wide variety of rural-urban environments and different types of housing in England. The NeDeCC was used to measure the residential environment of each participant, and significant bivariate relationships with wellbeing variables were identified.

**Results:**

The NeDeCC was found to have convincing face and construct validity and good inter-rater and test/retest reliability, though it would benefit from use of digital data sources such as Google Earth to eliminate the need for on-site survey. The significant relationships found in the study suggest that there may be characteristics of residential environments of potential relevance for older people's lives that have been overlooked in research to date, and that it may be worthwhile to question some of the assumptions about where and how older people want to live (e.g. villages seem to be positive). They also point to the importance of considering non-linear relationships.

**Conclusions:**

The NeDeCC provides the basis for generation of evidence-based design guidance if it is used in prospective controlled studies or 'natural experiments' in the future. Ultimately, this will facilitate the creation of better places for ageing in place.

## Background

With the recent shift in health provision away from a medical model to a focus on health promotion, increasing attention is being paid to the role of the built environment, particularly within residential areas [1-4]. According to Jackson, 'we now realise that how we design the built environment may hold tremendous potential for addressing many of the nation's greatest public health concerns' [5]. Much of the research in this area has concentrated on the links between neighbourhood characteristics and physical activity or obesity [6,7], though there has also been interest in respiratory health [8,9] and mental health [10]. Another key area of research is the impact of low carbon design on winter deaths [11].

There are good reasons for investigating the role of the built environment in healthy ageing. As in clinical medicine it is recognised that people need to receive different medical treatments at different stages of life, so it can be argued that non-medical determinants of health will differ in their impacts over the life-course [12]. Longevity is a pressing issue for public health, not least because of the greater likelihood of frailty and disability in older age [13]. Consideration of the built environment is particularly pertinent for older people: as they age, they are likely to spend more time in their home and community environments, and declining health and functional status can make them more susceptible to barriers in them [14]. Escalating care costs and evidence that the majority of older people prefer to remain living in their own homes has led to widespread adoption of 'ageing in place' policies [15].

For ageing in place to work well, housing and neighbourhood environments need to facilitate older people's independence and wellbeing. According to Liu et al [16], recent research suggests that wellbeing in later life is closely related to the physical environment, which is an important mediator of ageing experiences and opportunities. The physical character of the neighbourhood in particular seems to have a significant impact on the mobility, independence and quality of life of older people living in the local community [17]. The idea of age-friendly communities was developed by the World Health Organisation through their Global Age Friendly Cities Project launched in 33 cities in 2005. There are alternative names for such communities: for example, they tend to be called 'liveable communities' in the US and 'lifetime neighbourhoods' in the UK [18]. The features of each are similar though they differ in terms of the emphasis given to physical or social elements.

There is a common view, now apparent in UK planning policies and elsewhere, that older people need higher-density homes (usually apartments) in urban locations. This is based on several assumptions: older households prefer less space and freedom from the burden of looking after a large house and garden [19]; being in urban locations allows older people easy access to public transport, shops, health facilities and other amenities at a time when they may no longer be able to drive or afford to own a car [20]; and living in higher-density, urban locations provides older people with greater opportunities for social interaction, involvement in the local community and stimulation/interest [21]. It is important to investigate whether policies intended to address the needs of older people are in fact delivering benefits to them.

What is lacking at the moment, in terms of both research and practice, is an integration of the health and built environment areas of expertise [22,23]. Recognition of the relationship between the built environment and health opens up new avenues for health-promoting interventions, but little progress can be made without evidence of the role of different aspects of the built environment [24]. There are many methodological challenges in conducting research on the built environment and health and wellbeing, and these are fairly well documented: e.g. impracticalities in carrying out trials or controlled experiments, the need to account for residential self-selection or drift, and difficulties in controlling for other influences on wellbeing [25,26].

From a built environment perspective, perhaps the most significant shortcoming in research to date is that it is often difficult to translate the findings into practice - i.e. to know how the built environment should be changed or designed differently to optimise wellbeing [27]. This is largely because of how the built environment has been measured:

• Often it is treated as a 'black box', despite comprising a huge number of individual elements: for example, a neighbourhood environment is unique in terms of its street layout, street design, amount of greenery, presence of street furniture, and the type and variety of buildings within it (to name but a few different elements). In order to know how best to intervene, built environment practitioners (designers, developers etc.) need to know not just whether the built environment makes a difference but also which elements or characteristics are important [28].

• Measures are often subjective - i.e. ratings of quality by residents or researchers [e.g. [29]]. Subjective measures are prone to recall error, and same-source bias (e.g. unhappy people are likely to rate their environment more negatively) [30]. Further, they have the same limitations as those described above - i.e. they are difficult to translate into practice (what is a 'better quality' environment?). Lin and Vernez Moudon [31] found that objective measures of the built environment had stronger associations with amount of walking than subjective measures of the same attributes. Several tools for measuring built environments take the form of an audit or evaluation rather than a simple description - 'walkability' tools are examples of these [32].

• Measures are often a combination of physical and social characteristics, including condition of buildings and streets and prevalence of vandalism, litter and graffiti (e.g. the REAT instrument [33]) [34,35]. Researchers are right to believe all these characteristics play a role in people's wellbeing, but from the perspective of a built environment practitioner, they need guidance on those elements over which they have some control.

• Measures are collected for a spatial unit, such as a 'lot' or block face, rather than for an individual [36] - as Clarke and Nieuwenhuijsen [37] argue, this may not represent the experiential environment of the respondent from whom health or wellbeing data is being collected. Wood et al [38] measured the residential environment of individuals, but only on four attributes.

The ultimate goal in terms of investigating the impact of the residential environment on healthy ageing might be an experiment or trial involving the evaluation of an intervention, or a prospective controlled study investigating outcomes for older people moving into different settings. However, while there is some evidence that the built environment makes a difference in healthy ageing, there is little understanding of which individual design characteristics are important. The use of objective measures of the built environment has been growing in recent years, mainly through research on obesity [39]. These measures usually include characteristics such as street connectivity, block size, land use mix or presence of 'destinations', development density, presence and width of footpaths, topography, and sprawl [40-43]. Less commonly, studies address street surveillance, street lighting, presence of trees, urban-rural location type, traffic speed, presence of traffic control devices and public transportation infrastructure provision [44,45].

Studies of the built environment in relation to mental health, stress and social capital use some of the measures above but additional ones related to the more immediate home environment, for example dwelling form/type, storey height of housing, floor level of dwelling, characteristics of the street/home interface (e.g. setback of home, presence of porches/stoops) [46-50]. Purciel et al [51] have attempted to measure more subtle urban design constructs, including imageability, enclosure, human scale, transparency and complexity, using objective data from GIS. This inventive work is potentially of great relevance to urban designers, but the difficulty is ensuring the objective measures are a true representation of these constructs, and so far they have been developed for spatial units (block faces) rather than individuals. Brown et al [52] at the University of Miami have devised the Built Environment Coding System, which has many strengths. It measures objectively a wide range of urban design attributes. It is, however, limited in use because, again, it obtains measures for the spatial unit of 'lots' rather than individuals' residential environments.

The purpose of the research reported here was to develop a tool which measures a wide range of urban design characteristics within an individual's residential environment, and to test this tool in a preliminary study, in order to identify potential predictors of older people's wellbeing that are worthy of investigation in future research.

## Methods

Using a sample of 200 older people living in a wide range of locations in the UK, various 'place-related' aspects of wellbeing were measured through in-depth interviews (guided by a questionnaire). Urban design characteristics of their neighbourhoods were measured objectively using a newly-designed and tested tool, the NeDeCC (Neighbourhood Design Characteristics Checklist).

### The Neighbourhood Design Characteristics Checklist (NeDeCC)

The NeDeCC develops previous work by one of the authors (EB) to measure or describe discrete housing areas using a Built Environment Site Survey Checklist (BESSC) [53]. The new tool is based on the following principles:

1. It comprises objective measures of the built environment, which could be termed 'descriptions' rather than evaluations;

2. It attempts to measure a wide range of built environment characteristics within an individual's experiential residential environment, but only those that are modifiable in practice;

3. The tool is designed to measure the residential environment of an individual rather than a predetermined spatial or geographical unit;

4. The measures, though based around an individual residence, extend from their front door to the street on which they live and the wider neighbourhood beyond;

5. The tool is applicable across the full range of residential environments on the rural-urban spectrum or whatever the cultural/international context;

6. It is suitable for use by non-built environment researchers, being easy to apply without the need for specialist equipment or software.

Reference was made to standard urban design texts and design guidance documents to ascertain the range of characteristics to be measured [e.g. [54-60]]. Research literature was then reviewed to identify when and how any of these characteristics had been measured in previous studies. From this, response codings were devised for each naturally categorical urban design characteristic. The tool is in the form of checklist containing 25 individual items, each representing an individual characteristic of a research participant's residential environment. See Additional file 1 for a list of NeDeCC items. There are three parts to the checklist: items related to an individual residence (4 items); those related to the street on which the residence is located (9 items); and those related to the wider neighbourhood around the residence (12 items). The majority of items are coded through on-site survey by a non-specialist researcher; a few items are coded through map analysis.

To measure an individual's residential environment using the NeDeCC, a map (e.g. Ordnance Survey in the UK) was obtained first, using their residential address or postcode as the reference point. A scale of about 1:1250 was found to be appropriate as this shows facilities, services, open space and trees. The extent of the individual's residential environment was defined and marked on the map as everything within a 300 m radius from their home that could be accessed on foot (being aware that maps are not always up to date). Areas not accessible to pedestrians due to physical barriers (e.g. rivers and railway lines) were excluded from the survey. The 300 m radius was derived from an estimate of the average distance older people would be able to walk comfortably from their homes, based on 10 minutes' walking time and findings that older people walk more slowly than the average fit male adult [61]. Obviously, this radius could be changed, depending on the target population for the research. All surveys were carried out between 10 am and 3 pm weekdays or during daylight hours at weekends (to avoid peak hours, school runs etc.). The map measurements were obtained first so that anything that was not clear from the map could be checked during the survey.

As a measurement tool the NeDeCC has good face-value validity; each item is measuring a distinct, easily-identifiable objective physical quality (as opposed to a broader concept requiring a substantial degree of interpretation). The construct validity of the NeDeCC, i.e. how far it adequately measures an individual's residential environment, was maximised by a) including only objective built environment measures (no subjective concepts such as quality, condition or attractiveness which are difficult to operationalise) and b) including a broad range of items identified from focus groups and literature. This addressed weaknesses in previous measures of the built environment which included only single or ill-defined elements. None of these individual items were combined within a composite measure. A test of construct validity is how far the measures are associated with those you would expect them to be, from theory and previous research. For built environment measures, this is somewhat difficult to examine because of the paucity of research and evidence to date on individual elements of the environment. However, there is support for the construct validity of the NeDeCC because in general the associations found to be significant between individual built environment items and aspects of wellbeing were as theorised (not so much in ageing literature but in general literature on environment and wellbeing). For example, wellbeing was associated with the amount of greenery in the neighbourhood, supporting many other studies; and older people felt safest if they lived in villages, as found in previous work.

The tool was piloted to test its usability. From this, the wording of items and their responses were refined for the purposes of clarity. Then, to check the inter-rater reliability of the tool, ratings were completed by two researchers for a number of individuals living in different neighbourhoods. The inter-rater reliability was found to be satisfactory for all the items (kappa ≥ 0.6 for the categorical items). It was possible to ensure a level of test/retest reliability, even though this is less of an issue for a built environment measure (some of the human error such as recall error is removed). As the tool was used within a 300 m radius of each participant's home and participants were sometimes clustered within geographical areas, there was some overlapping of areas measured. The team found that overlapping areas were rated the same by researchers on the different occasions they collected the data.

### Measuring wellbeing

According to Masotti et al [62], a neighbourhood environment can be made healthier for older adults by changing characteristics to increase activity, create a sense of community, and hence benefit wellbeing. Commentators in the field have argued that there is a lack of understanding of the pathways of influence of the built environment on health and wellbeing in older age - i.e. little is known about how the built environment plays a role [63,64]. For this reason, it was decided that, in order to identify characteristics of urban design that are worthy of deeper investigation, it was appropriate to examine links with multiple wellbeing constructs. Fourteen constructs were selected by pinpointing from the literature and a series of focus groups with older people (seven groups, 38 participants) aspects of older people's health, wellbeing, satisfaction and quality of life that seem most likely to be affected by the built environment. Together, these could be termed 'place-related' wellbeing constructs. They fall into three general categories: those that are functional in nature; those that are social; and those that are emotional (see below).

*Functional place-related wellbeing (6 items): *amount of independence participants feel they have in life; perceptions of safety from traffic and non-motorised traffic (e.g. bikes, skaters); incidence of falling outside; perceived noise problems; perceived air quality.

*Social place-related wellbeing (4 items)*: perceived community spirit; extent of social interaction; perceptions of safety from crime (before and after dark).

*Emotional place-related wellbeing (4 items)*: self-rated quality of life; satisfaction with the neighbourhood as a place to live; perceived attractiveness of the neighbourhood; enjoyment of trips in local neighbourhood.

### Research sample

Wellbeing and neighbourhood characteristics were measured for a sample of 200 older people. This was considered the maximum sample size possible given the research timeframe and the need to conduct detailed surveys of each participant's residential environment. Older people were defined as those aged 65 and over, in line with previous studies.

Older people were recruited from a wide range of location types, in Oxfordshire, Gloucestershire and Greater Manchester, including city/town centres, urban districts, suburbs/edges and villages, representing contrasting types of environment. Additionally, the sample was selected to provide a mix of older people living in age-specific housing (including sheltered housing and retirement homes) and 'ordinary' housing, and to provide a mix of social and private housing. Recruitment was carried out through two methods: for age-specific housing, the researchers approached housing associations and private developers, including those linked to the project as non-academic collaborators; people living in ordinary housing were recruited through older people's organisations (e.g. lunch clubs, church groups). Ongoing recruitment was reviewed to identify gaps in terms of different social groups (e.g. ethnic mix, age), urban-rural locations or housing types, and further recruitment was targeted accordingly. Characteristics of the sample are summarised in Tables 1 and 2. The economic status of participants' neighbourhoods was determined using the Office of National Statistics (ONS) Index of Multiple Deprivation (IMD), which enables the identification of deprivation ranks at the postcode level. IMD ranks for participants' neighbourhoods ranged from 20 to 32, 455 with 20 having the highest deprivation.

**Table 1:** Characteristics of older people in the sample

Variable	Category	% of participants
Gender	Women	59.0
	Men	41.0
Age group	65-74	43.5
	75-84	43.0
	80+	13.5
Socio-economic classification	I Professional	15.5
	II Managerial	40.0
	IIIN Skilled - non manual	16.5
	IIIM Skilled - manual	14.5
	IV Partly skilled	11.0
	V Unskilled	2.5
Self-rated health	Excellent	12.0
	Very good	34.0
	Good	24.5
	Fair	19.5
	Poor	10.0
Specific health problems	None	16.5
	Visual impairments	26.0
	Hearing impairments	35.5
	Memory problems	24.0
	Mobility problems	59.5
	Other	6.0
Activities limited by health problems	Yes	51.5
	No	48.5
Activities limited the most by:	Visual impairments	3.5
	Hearing impairments	2.0
	Memory problems	1.0
	Mobility problems	44.5
	Other (heart and breathing problems)	0.5
Use of mobility aids when out	None	64.5
	Walking aid	28.0
	Wheelchair	2.5
	Help from another person	1.5
	Mobility scooter	8.0
Forms of transport used the most often	Walk	85.0
	Bicycle	3.0
	Bus	56.0
	Drives own car	51.0
	Driven by someone else	19.5
	Train	10.0
	Taxi	16.5
	Dial-a-ride	4.5
	Mobility scooter	7.0
Living arrangements	Alone	54.5
	With partner	41.5
	With others	4.0
Length of time living in neighbourhood	< 1 year	3.5
	1-10 years	38.5
	> 10 years	58.0
Tenure	Owner-occupier	62.5
	Renting from Housing Association	37.5

**Table 2:** Index of Multiple Deprivation rankings for research participants (using their postcodes)

IMD Ranks	% of participants
≤1000	6.5
1001-5000	9.0
5001-10000	16.5
10001-15000	10.5
15001-20000	12.0
20001-25000	19.5
25001-30000	19.5
30001+	6.5

### Analysis

The responses to each built environment measure arising from the NeDeCC were first investigated to determine the types of neighbourhoods that were present. Correlations between wellbeing variables were also examined to determine whether distinct constructs were being measured, or whether the potential existed to combine items into a smaller number of composite measures. Two sets of tests of independence were then performed. The first set compared housing type to responses to every other built environment measure; the second tested relationships between each of the 25 individual built environment characteristics and each of the 14 functional, social and emotional place-related wellbeing constructs. These analyses used a combination of Pearson chi-square tests where both variables were categorical and at least one was nominal in form; rank correlations where both variables were at least ordinal; and Kruskal Wallis tests where one variable was nominal and the other was of continuous or discrete type. A significance level of p < 0.05 was applied to all tests, adjusted for the number of tests being run by a Bonferroni correction to mitigate the risks of type I errors [65]. As such, for tests between housing type and built environment measures, a p value of less than 0.05/23 = 0.0022 was required to reject the null hypothesis of independence in favour of the alternative of a significant association. For tests between each pair of built environment measures and wellbeing dimensions, a p value of less than 0.05/336 = 0.00015 was required to similarly reject the null hypothesis.

## Results and discussion

Table 3 summarises the differences between the residential environments of participants according to the individual characteristics measured by the NeDeCC, and whether or not they were significantly associated with housing type. As the sample was relatively small and non-random it is not possible to generalise from this about the character of places in which older people are ageing. However, the findings illustrate a wide spread in terms of the different characteristics.

**Table 3:** Characteristics of the residential environments of participants

		% of participants or median/range	Significant association with housing type?
Type of participant's dwelling	General private housing	39.5	NA
	General social housing	15.5	
	Sheltered private housing	23.0	
	Sheltered social housing	22.0	
Form of participant's dwelling	Detached house	8.5	√
	Detached bungalow	5.5	
	Semi-detached house	12.5	
	Semi-detached bungalow	6.0	
	Terraced house	22.0	
	Terraced bungalow	4.0	
	Converted flat	2.5	
	Purpose built low-rise flat	27.0	
	Purpose built high-rise flat (3+ storeys)	12.0	
Height of participant's dwelling	3+ storeys	29.0	√
	2 storeys	55.0	
	1 storey	13.0	
Approximate age of participant's dwelling	Pre-1914	7.0	√
	1914-1939	15.0	
	1940-1969	13.5	
	1970-1989	35.5	
	1990+	27.5	
Type of participant's street	Main road in city/town	23.5	√
	High street in city, town or village	2.0	
	Residential street/square	35.0	
	Residential cul-de-sac	33.0	
	Open access mews/courtyard	3.0	
	Rural through street	2.5	
	Rural side street/lane	0.5	
	Other	0.5	
Shape of participant's street	Straight	41.5	√
	Gentle curve	21.5	
	Serpentine	9.5	
	Tight curve	2.0	
	Loop	5.5	
	Cul-de-sac	18.5	
	Crescent	1.0	
	Square	0.5	
Topography of participant's street	Flat	53.5	X
	Gently sloping without steps	28.5	
	Gently sloping with steps	0.5	
	Steep (over 5% or 1 in 20)	11.5	
	without steps	0.5	
	Steep (over 5% or 1 in 20) with steps	5.5	
	Undulating		
Pedestrian/traffic segregation on participant's street	No footway - roadway only	8.5	√
	Shared surface e.g. Home Zone	1.0	
	Delineated non-raised footway	1.0	
	Raised footway	80.5	
	Raised footway/vegetation	7.5	
	Raised footway divided between pedestrians and cyclists	0.5	
	Raised footway/cycle lane on road	0.5	
	Pedestrianised	1.0	
Extent of "eyes on the streets" on participant's street	Large amount	34.0	√
	Moderate amount	46.5	
	Small amount	14.5	
	Great variety along the street	5.0	
Extent of variety of built form on participant's street	Generally uniform	47.0	√
	Fairly varied	31.0	
	Greatly varied	22.0	
Size of block participant's house is situated in	Very large block (≥250 m)	60.0	√
	Large block (151-249 m)	20.0	
	Medium block (91-150 m)	11.0	
	Short block (≤ 90 m)	4.0	
	Not applicable	5.0	
Predominant setback of buildings from street	Zero setback	3.5	√
	≤2 m	10.5	
	> 2 m/< 5 m	44.0	
	≥5 m	19.5	
	Varied	22.5	
Residential location	Major city/town centre	7.5	√
	Major city/town district	22.5	
	Major city/town suburban/edge	26.0	
	Large town centre	6.5	
	Large town suburban/edge	15.0	
	Small town	9.5	
	Village	13.0	
Predominant block size within 300 m radius	Very large blocks (≥250 m)	31.0	√
	Large blocks (151-249 m)	23.0	
	Medium blocks (91-150 m)	14.5	
	Short blocks (≤ 90 m)	26.0	
	Varied	0.5	
	Not applicable	5.0	
Predominant street pattern within 300 m radius	Regular geometric grid	9.0	√
	Distorted grid	35.0	
	Curvilinear (looped)	15.0	
	Cul-de-sac (tree pattern)	13.0	
	Radial	8.0	
	Ribbon	6.5	
	No discernible pattern	13.5	
Predominant mix of uses within 300 m radius	Residential	22.0	√
	Residential with occasional other uses	39.5	
	Fine grain mix of residential and non-residential usesClusters of residential and	14.5	
	non-residential	24.0	
Density of built-up area within 300 m radius	Very high density	2.0	√
	High density	24.5	
	Moderate density	50.5	
	Low density	15.5	
	Very low density	7.0	
	Mixed	0.5	
General extent of natural surveillance within 300 m radius	Large amount	19.0	√
	Moderate amount	57.5	
	Small amount	11.0	
	Great variety of level of surveillance throughout the neighbourhood	12.5	
General level of legibility within 300 m radius	Very large amount	7.5	x
	Large amount	30.0	
	Moderate amount	38.0	
	Small amount	21.5	
	Very small amount	3.0	
General amount of traffic within 300 m radius	Heavy	25.0	√
	Medium	49.5	
	Light	25.5	
General amount of greenery within the 300 m radius	Very large amount	7.0	√
	Large amount	37.5	
	Moderate amount	36.0	
	Small amount	17.5	
	Very small amount	2.0	
Motorised traffic level (number of vehicles over 2 minutes) in participant's street	Median	1	√
	Minimum	0	
	Maximum	55	
Total amount of open space within 300 m radius (ha)	Median	3.39	X
	Minimum	0.00	
	Maximum	29.10	
Total number of junctions within 300 m radius	Median	23	√
	Minimum	3	
	Maximum	332	

Furthermore, nearly all the built environment measures were significantly associated with housing type (only topography, legibility and amount of open space were unrelated). This suggests that older people are living in very different types of environments depending on whether their home is sheltered accommodation or general housing, and/or whether it is private or social (i.e. rented from a housing association or not-for-profit developer). Moving between these types of housing could therefore have substantial implications for the character of environments older people inhabit.

The second set of analyses tested relationships between the 14 wellbeing variables and 25 built environment variables. The analysis was hampered by a lack of spread for many of the wellbeing responses, particularly the emotional ones; for instance, self-rated quality of life, satisfaction with the neighbourhood, and enjoyment of trips in it. In general, the older people in the sample were very positive about all aspects of their wellbeing. However significant relationships with wellbeing variables were found for a number of different built environment characteristics. As the study is exploratory, the exact nature of these relationships is unclear, but they are summarised in tabular form in Additional file 2.

The table shows a spread of significant relationships among the variables. These should be interpreted with caution, particularly as the analyses did not control for background variables - i.e. it is likely that certain types of area are inhabited by certain types of people, so the relationships may simply reflect relationships between personal characteristics and wellbeing constructs. Further, the lack of sensitivity in the wellbeing measures may have masked differences for people in different environments. The dimensions of wellbeing with the greatest number of associations were: perceptions of safety from non-motorised traffic (bicycles and skateboards); perceptions of safety after and before dark; and self-rated quality of life. The built environment measures found to have the greatest number of significant associations were: type of housing; residential location on the urban-rural spectrum; built-up density; and number of junctions/intersections within 300 m radius. No associations were found for extent of 'eyes on the street' or motorised traffic level on particpant's street. Several measures were found to have only one significant association: age of housing; shape of street; size of block; and extent of natural surveillance within 300 m radius. However, this does not necessarily mean that these aspects of the built environment are unimportant for older people's wellbeing - if investigated further, they may be found to be strongly associated with wellbeing, even after controlling for background variables.

Overall, the findings suggest that in future research it would be worthwhile investigating a wide range of different dimensions of urban design and residential environments, including those not commonly addressed (e.g. street shape, street pattern), though not necessarily within one study. It is also worth investigating a wide range of pathways for the influence of the built environment. Though conclusions cannot be drawn from the superficial analysis in this study, it is notable that all the wellbeing constructs were found to be associated with at least one built environment measure. To delve a little deeper into the findings, some of the stronger associations were examined more carefully - the preliminary findings are reported below.

*Amount of greenery*: older people living in more green areas reported greater satisfaction with their neighbourhood as a place to live; they were also more likely to perceive it as attractive - clearly, the two may be related.

*Density*: older people living in higher-density areas tended to feel less safe from non-motorised traffic (cyclists and skaters) while those in moderate-density areas tended to feel less safe from motorised traffic (there was a squared relationship here, where people in both low- and high-density areas felt safer); in terms of social wellbeing constructs, older people in higher-density environments tended to feel less safe from crime, both before and after dark.

*Location*: older people living in villages were most likely to rate their environment as very attractive, perhaps because they are greener; perceived safety before dark was found to be better in villages and small towns than cities and large towns, while perceived safety after dark was found to be best in villages and suburbs of large towns and worst in districts and centres of large and major towns/cities; perceived safety from non-motorised traffic was found to be worst among older people living in districts of major towns/cities and best among those living in villages and small towns.

*Street pattern*: perceived safety from non-motorised traffic was found to be worst among older people living in distorted grid layouts and best in curvilinear (looped) layouts.

*Block size*: in areas with very large blocks, older people were more likely to feel safe from non-motorised traffic, while those in areas with medium-sized blocks were likely to feel least safe.

*Setback of dwellings from the street*: older people were more likely to report feeling safe from non-motorised traffic if they lived in areas where the buildings were set well back from the street, probably because this provides a buffer zone between the front door and cyclists or skaters on the pavement.

*Street topography*: older people living on flat streets were less likely to report safety from non-motorised traffic, perhaps because there are more cyclists and skaters in flat areas.

The findings taken as a whole suggest that it is worth questioning some of the assumptions about how and where older people want to live - e.g. that they are better off in higher-density, urban areas. Further, it should be noted that the relationships between wellbeing and built environment characteristics may not be linear. Here, it was found that older people reported positive feedback in both high- and low-density neighbourhoods and negative feedback in moderate-density neighbourhoods (a curvilinear relationship). The potential for non-linear relationships should be taken account in future research - there may well be characteristics that are positive up to a point beyond which they become problematic. This is important information for designers and other providers of the built environment.

Another key finding to draw from the research is the importance of meaningful calibration of measures. It is necessary not only to measure each relevant aspect of urban design within a residential environment but also to measure this well, using categories that allow adequate correlation with wellbeing outcomes.

## Conclusions

This study shows that it is possible to measure objectively a wide range of urban design characteristics, reflecting the nature of residential environments within which older people live. It has developed and tested a new tool, the NeDeCC, which can be used in further research. It can be used anywhere, in rural as well as urban locations, and in any part of the world, as the measures are independent of cultural context. The development of these measures provides a good basis for rigorous investigation of the role of the built environment in health and wellbeing from which it is possible to generate guidance for designers and providers of the built environment - architects, urban designers, housing developers, social housing providers and so on. There is scope for improving and developing the built environment tool. The authors are currently adapting it for completion through remote measurement, using digital maps and Google Earth Street View, eliminating the need for on-site survey. This saves time and resources and allows its use for large sample sizes. Other researchers have found these digital sources to be reliable alternatives to on-site survey [66,67].

Clearly, the potential of the tool would be maximised through its use in more sophisticated study designs: for example, recording changes to the built environment in 'natural experiments', prospective controlled studies or intervention trials, where background variables are controlled and account is taken of residential self-selection or drift. A major shortcoming of the research reported here was inadequacy of the wellbeing constructs that were used. The response was overwhelmingly positive, albeit partly because of the focus on older people; this degree of range restriction reduced statistical power, making it difficult to detect relationships with NeDeCC dimensions. More or different relationships may be found if better measures of wellbeing are used in future research. A validated scale such as WEMWBS (Warwick and Edinburgh Mental Wellbeing Scale), which provides a continuous measure based on a number of different parameters, would improve the power of the research.

There is little doubt that one of the most important public health challenges today is the loss of wellbeing in society [68]. Increased economic growth in the US, Europe and Australasia since the 1970 s has not been accompanied by improvements in wellbeing, which may now be declining [69]. In the pursuit of wellbeing in society it is necessary to look more closely at non-medical determinants [70]. An important one of these is the built environment. The emerging science of health promotion requires a multi-disciplinary approach. This is easier said than done, because while built environment research is generally underdeveloped, health research has strong traditions and paradigms that do not easily accommodate complex interventions. This research is an attempt by built environment researchers to carry out a genuinely trans-disciplinary study in this field, in order to generate preliminary indications of physical characteristics of neighbourhoods worthy of deeper investigation. It contributes built environment measures that will facilitate the generation of valuable evidence in the pursuit of ageing in place. Design of environments for older people is rarely based on empirical evidence; Kendig [71] notes that the kinds of housing which markets, governments and community groups make available for older people reflect deeply seated images and in some cases ageism. Although there is now a growing body of evidence for healthcare buildings, it is almost non-existent for the design of housing, streets and neighbourhoods [72]. This study contributes to what will hopefully be a burgeoning field of study, of potentially high impact. Ultimately, the aim is to find out how to create and adapt environments to facilitate the wellbeing and positive health of all people, throughout their lives.

## Competing interests

The authors declare that they have no competing interests.

## Authors' contributions

EJB was the principal investigator for the study - i.e. she developed the ideas and study design and methods and managed the research team collecting and analyzing the data. EJB also contributed to data analysis and drafted the manuscript. LM helped to develop the research design and NeDeCC tool and undertook most of the data acquisition and analysis. She also helped to draft the manuscript. CS provided the statistical expertise for the study - he was not involved in developing the NeDeCC tool and study design but undertook all aspects of analysis and helped prepare the manuscript. All authors read and approved the final manuscript.

## Authors' information

EJB (MA Cantab DipArch DipUD PhD) is Professor of Sustainable Building Design and Wellbeing and founder director of the WISE (Wellbeing in Sustainable Environments) research unit at the University of Warwick. Having qualified as an architect and urban designer, EJB took up a research career, with the aim of developing an evidence base for architectural practice. Her research investigates the social aspects of sustainability and how the built environment (architecture and urban design) influences people's wellbeing, quality of life and mental health. She has particular expertise in ageing research, including dementia-friendly design. She has also devised innovative tools and methods for obtaining objective measures of the built environment. EJB is now seeking to promote design for wellbeing in the built environment through the development of new cross-disciplinary courses.

LM (BA Hons MPhil PhD) is co-founder of the WISE (Wellbeing in Sustainable Environments) research unit in the School of Health and Social Studies, University of Warwick. Her background is in social sciences, planning and inclusive urban design. Her research interests lie in how the design of the built environment, such as care settings, housing, urban form and streets, influences people's health and wellbeing and ability to lead independent, active lives. She has particular expertise in researching the design needs of older people and people with dementia.

CBS is the statistician at the Institute of Work Psychology at the University of Sheffield. He has published across a wide range of social science disciplines, and is particularly interested in the use of statistical methods to support and add rigour to research in areas where advanced quantitative analysis would typically be considered an anathema.

## Pre-publication history

The pre-publication history for this paper can be accessed here:

http://www.biomedcentral.com/1471-2458/11/839/prepub

## Supplementary Material

Additional file 1 **Neighbourhood Design Characteristics Checklist (NeDeCC)**. Items and categorical responses included in the NeDeCC instrument. Click here for file

Additional file 2 **Significant relationships between built environment and wellbeing variables**. This table shows significant relationships between built environment and wellbeing variables. Click here for file
