# Supplementary material for: Good places for ageing in place: development of objective built environment measures for investigating links with older people's wellbeing
Source: BMC Public Health. 2011 Nov 1;11:839. doi: 10.1186/1471-2458-11-839 (PMC3214925; doi:10.1186/1471-2458-11-839)
Supplement: Additional file 1 — Neighbourhood Design Characteristics Checklist (NeDeCC). Items and categorical responses included in the NeDeCC instrument. [file 1471-2458-11-839-S1.DOCX]

**Neighbourhood Design Characteristics Checklist (NeDeCC)**

| **A. PARTICIPANT’S DWELLING** | | |
| --- | --- | --- |
| **A1** | **Type of housing** | 1. General private housing 2. General social housing 3. Sheltered private housing 4. Sheltered social housing |
| **A2** | **Form of dwelling** | 1. Detached house 2. Detached bungalow 3. Semi-detached house 4. Semi-detached bungalow 5. Terraced house 6. Terraced bungalow 7. Maisonette 8. Converted flat 9. Flat above shop/office 10. Purpose built low-rise flat 11. Purpose built high-rise flat (3+ storeys) 12. Other |
| **A3** | **Height of dwelling** | 1. 3+ storeys 2. 2 storeys 3. 1 storey |
| **A4** | **Approximate age of dwelling** | 1. Pre-1914 2. 1914-1939 3. 1940-1969 4. 1970-1989 5. 1990+ |
| **B. PARTICIPANT’S STREET** | | |
| **B1** | **Street type**  6) & 7) Mews/courtyards have dwellings around a central space with a pedestrian/vehicle shared surface | 1. Main road in city/town 2. High street in city, town or village 3. Avenue 4. Residential street/square 5. Residential cul-de-sac 6. Gated mews/courtyard 7. Open access mews/courtyard 8. Rural through street 9. Rural side street/lane 10. Other |
| **B2** | **Street shape**  Tight curve = 70-90 degrees | 1. Straight 2. Gentle curve 3. Serpentine 4. Tight curve 5. Loop 6. Cul-de-sac 7. Crescent 8. Square 9. Other |
| **B3** | Pedestrian/traffic segregation | 1. No footway - roadway only 2. Shared surface e.g. Home Zone 3. Delineated non-raised footway 4. Raised footway 5. Raised footway/vegetation 6. Raised footway divided between pedestrians and cyclists 7. Raised footway/cycle lane on road 8. Pedestrianised |
| **B4** | Street topography 3) & 5) Where steps are part of the footway | FlatGently sloping without stepsGently sloping with stepsSteep (over 5% or 1 in 20) without stepsSteep (over 5% or 1 in 20) with stepsUndulating |
| **B5** | Extent of “eyes on the streets” Count first 3 storeys only  1) Doors/windows facing the street at regular intervals  3) Hardly any doors/windows, mainly blank walls, facades etc | 1. Large amount 2. Moderate amount 3. Small amount 4. Great variety along the street |
| **B6** | Extent of variety of built form e.g. materials, heights, styles, shapes, fenestration, architectural | 1. Generally uniform 2. Fairly varied 3. Greatly varied |
| **B7** | **Size of block participant’s house is situated in**  A block perimeter can be delineated by roads and/or pedestrian-only public footways e.g. alleys | 1. Very large block (≥250m) 2. Large block (151-249m) 3. Medium block (91-150m) 4. Short block (≤ 90m) |
| **B8** | Motorised traffic level Count motorised traffic moving in both directions outside participant’s house for 2 minutes. Also note general speeds |  |
| **B9** | **Predominant setback of buildings from street**  From building perimeter to footway | 1. Zero setback 2. ≤2m 3. >2m/<5m 4. ≥5m 5. Varied |
| **C. PARTICPANT’S NEIGHBOURHOOD** | | |
| **C1** | **Residential location** | Major city/town centre  1. Major city/town district 2. Major city/town suburban/edge 3. Large town centre 4. Large town suburban/edge 5. Small town 6. Village |
| **C2** | **Predominant block size within 300m radius**  5) Where there is no predominance of one particular size category | 1. Very large blocks (≥250m) 2. Large blocks (151-249m) 3. Medium blocks (91-150m) 4. Short blocks (≤ 90m)   5) Varied |
| **C3** | **Predominant street pattern within 300m radius** | 1. Regular geometric grid 2. Distorted grid 3. Curvilinear (looped) 4. Culs-de-sac (tree pattern) 5. Radial 6. Ribbon 7. No discernible pattern |
| **C4** | **Total number of junctions within 300m radius** |  |
| **C6** | **Total amount of open space within 300m radius (ha)** |  |
| **C7** | Predominant mix of use within 300m radius 3 = interspersed within the block horizontally and vertically  4 = horizontal mix in discrete zones block to block | 1. Residential 2. Residential with occasional other uses 3. Fine grain mix of residential and non-residential uses 4. Clusters of residential and non-residential |
| **C8** | **Density of built-up area within 300m radius**  1 = mainly 4+ storey terraced forms  2 = mainly 2-3 storey terraced forms  3 = mainly 2 storey semi-detached forms  4 = mainly 1-2 storey detached forms  5 = occasional 1-2 storey detached forms | 1. Very high density 2. High density 3. Moderate density 4. Low density 5. Very low density 6. Mixed |
| **C9** | **General extent of natural surveillance within 300m radius**  e.g. streets and open spaces overlooked by entrances and windows  1) Doors/windows facing the street at regular intervals  3) Hardly any doors/windows, mainly blank walls, facades etc | 1. Large amount 2. Moderate amount 3. Small amount 4. Great variety of level of surveillance throughout the neighbourhood |
| **C10** | **General level of legibility within 300m radius**  Visual links to civic, historic and distinctive landmarks and structures, and to main routes and destinations | 1. Very large amount 2. Large amount 3. Moderate amount 4. Small amount 5. Very small amount 6. None |
| **C11** | **General amount of traffic within 300m radius** | 1. Heavy 2. Medium 3. Light |
| **C12** | **General amount of greenery within 300m radius**  To record a general impression of the greenery of the whole neighbourhood within the 300m radius so include front gardens, grass verges, trees and planting. | 1. Very large amount 2. Large amount 3. Moderate amount 4. Small amount 5. Very small amount 6. None |
