# Supplementary material for: Good places for ageing in place: development of objective built environment measures for investigating links with older people's wellbeing
Source: BMC Public Health. 2011 Nov 1;11:839. doi: 10.1186/1471-2458-11-839 (PMC3214925; doi:10.1186/1471-2458-11-839)
Supplement: Additional file 2 — Significant relationships between built environment and wellbeing variables. This table shows significant relationships between built environment and wellbeing variables. [file 1471-2458-11-839-S2.DOC]

***Significant relationships between built environment and wellbeing variables***

|  | Amount of independence in life | Perceptions of safety from motorised traffic | Perceptions of safety from non-motorised traffic | Incidence of falling outside | Perceived noise problems | Perceived air quality | Self-rated quality of life | Satisfaction with neighbour-hood as place to live | Attractiveness | Enjoyment of trips out | Community spirit | Social interaction | Perceptions of safety when outside before dark | Perceptions of safety when outside after dark |
| --- | --- | --- | --- | --- | --- | --- | --- | --- | --- | --- | --- | --- | --- | --- |
| Type of participant’s housing | *√* |  | *√* | *√* |  |  | *√* | *√* | *√* | *√* |  | *√* | *√* | *√* |
| Form of participant’s housing |  |  | *√* |  |  |  | *√* |  |  |  |  |  | *√* |  |
| Height of participant’s housing |  |  | *√* |  |  | *√* |  |  |  |  |  |  |  | *√* |
| Approximate age of participant’s housing |  |  | *√* |  |  |  |  |  |  |  |  |  |  |  |
| Type of participant’s street |  |  |  | *√* |  |  |  |  |  |  |  |  |  | *√* |
| Shape of participant’s street |  |  |  |  |  |  |  |  |  |  |  |  |  | *√* |
| Topography of participant’s street |  |  | *√* |  |  |  |  |  |  |  |  | *√* | *√* | *√* |
| Pedestrian/traffic segregation on participant’s street |  |  |  |  | *√* | *√* | *√* |  |  |  |  |  | *√* |  |
| Extent of “eyes on the streets” on participant’s street |  |  |  |  |  |  |  |  |  |  |  |  |  |  |
| Extent of variety of built form on participant’s street |  |  |  | *√* | *√* |  |  |  |  |  |  |  |  |  |
| Size of block participant’s housing is situated in |  |  | *√* |  |  |  |  |  |  |  |  |  |  |  |
| Predominant setback of housing from participant’s street |  |  | *√* |  |  |  |  |  |  |  |  |  |  | *√* |
| Residential location |  |  | *√* | *√* |  | *√* | *√* |  | *√* |  |  | *√* | *√* | *√* |
| Predominant block size within 300m radius |  |  | *√* |  |  | *√* |  | *√* | *√* |  |  |  |  |  |
| Predominant street pattern within 300m radius |  |  | *√* | *√* |  |  |  |  | *√* |  |  |  | *√* | *√* |
| Predominant mix of uses within 300m radius | *√* | *√* | *√* |  |  | *√* | *√* |  |  |  |  |  | *√* |  |
| Density of built-up area within 300m radius |  |  | *√* | *√* |  |  | *√* | *√* | *√* | *√* |  |  | *√* | *√* |
| General extent of natural surveillance within 300m radius |  |  | *√* |  |  |  |  |  |  |  |  |  |  |  |
| General level of legibility within 300m radius |  |  | *√* |  |  |  |  | *√* |  |  |  |  |  |  |
| General amount of traffic within 300m radius |  |  | *√* |  |  | *√* |  |  |  |  |  |  |  |  |
| General amount of greenery within 300m radius |  |  |  |  |  | *√* | *√* | *√* | *√* |  |  | *√* |  | *√* |
| Motorised traffic level on participant’s street |  |  |  |  |  |  |  |  |  |  |  |  |  |  |
| Total amount of open space in hectares within 300m radius |  | *√* |  | *√* |  | *√* |  | *√* |  |  |  |  |  |  |
| Total number of junctions within 300m radius |  |  |  | *√* |  |  | *√* | *√* | *√* | *√* | *√* | *√* |  |  |
